# Single-cell RNA transcriptome reveals the intra-tumoral heterogeneity and regulators underlying tumor progression in metastatic pancreatic ductal adenocarcinoma

**DOI:** 10.1038/s41420-021-00663-1

**Published:** 2021-11-03

**Authors:** Qianhui Xu, Shaohuai Chen, Yuanbo Hu, Wen Huang

**Affiliations:** 1 The Second Affiliated Hospital and Yuying Children’s Hospital of Wenzhou Medical University, Wenzhou, Zhejiang 325000 China; 2 Zhejiang University School of Medicine, Hangzhou, Zhejiang 310009 China

**Keywords:** Pancreatic cancer, Tumour heterogeneity

## Abstract

Pancreatic ductal adenocarcinoma (PDAC) is the most frequent and aggressive pancreatic tumor characterized by high metastatic risk and special tumor microenvironment. To comprehensively delineate the complex intra-tumoral heterogeneity and the underlying mechanism during metastatic lesions malignant progression, single-cell RNA sequencing (scRNA-seq) was employed. PCA and TSNE were used for dimensionality reduction analysis and cell clustering. Find All Markers function was used to calculate differential genes in each cluster, and Do Heatmap function was used to plot the distribution of differential genes in each cluster. GSVA was employed to assign pathway activity estimates to individual cells. Lineage trajectory progression was inferred by monocle. CNV status was inferred to compare the heterogeneity among patients and subtypes by infercnv. Ligand-receptor interactions were identified by CellPhoneDB, and regulons network of cells was analyzed by SCENIC. Through RNA-sequencing of 6236 individual cells from 5 liver metastatic PDAC lesions, 10 major cell clusters are identified by using unbiased clustering analysis of expression profiling and well-known cell markers. Cells with high CNV level were considered as malignant cells and pathway analyses were carried out to highlight intratumor heterogeneity in PDAC. Pseudotime trajectory analysis revealed that components of multiple tumor-related pathways and transcription factors (TFs) were differentially expressed along PDAC progression. The complex cellular communication suggested potential immunotherapeutic targets in PDAC. Regulon network identified multiple candidates for promising cell-specific transcriptional factors. Finally, metastatic-related genes expression levels and signaling pathways were validated in bulk RNA Sequencing data. This study contributed a comprehensive single-cell transcriptome atlas and contributed into novel insight of intratumor heterogeneity and molecular mechanism in metastatic PDAC.

## Introduction

Pancreatic ductal adenocarcinoma (PDAC) is one of the most malignant tumors with a 5-year survival rate of 8% according to annual cancer statistical reports [1, 2]. This plight is mainly attributed to the lack of reliable markers for early diagnosis, low radical excision rate, and chemoradiotherapy resistance of PDAC, and only 15–20% of newly diagnosed patients are eligible for surgical resection [3, 4].

The intrinsic transcriptomic characteristics and dynamic immunological heterogeneity significantly contributed to the tumor progression and sensitivity toward treatment. Mounting genomic research have revealed crucial gene mutations, such as KRAS driver mutation and frequent inactivation of TP53 tumor suppressors, were the main drivers of tumorigenicity of PDAC [5]. Mutations of these genes participated in specific biological processes, including Wnt/TGF-β pathways, DNA repair processes, and chromatin remodeling mechanisms [6]. It was well established that PDAC is one of the malignant cancers characterized by diverse immune microenvironment comprised of abundant subpopulations of immune cells. A primary reason for the high mortality rate of PDAC is the abundant extracellular matrixes, which challenged against drugs delivery leading to the therapeutic resistance [7]. Thus, it is highly desirable to reveal the mechanisms underlying the tumorigenesis and metastasis of PDAC, and to determine robust and promising molecular targets for individualised therapeutic strategy.

In our work, singe cell RNA-sequencing analysis was employed to present a comprehensive landscape of the transcriptomic profiles of 6236 qualified single cells from 5 liver metastatic PDAC lesions, further dissecting intra-tumoral heterogeneity and identify crucial factors during PDAC metastatic progression. A total of ten major cell subpopulations were determined and the molecular properties of malignant cells were characterized. In addition, PDAC metastatic lesion constituted of diverse malignant cell subtypes was characterized by highly heterogeneous. We further identified a list of novel gene expression changes that affected several known cancerous pathways. With trajectory analysis of tumor progression, the distant dissemination function becomes increased. The comprehensive atlas of the multicellular ecosystem of TME were delineated and potential transcriptional regulators network underlying tumor progression was established. In addition, metastasis-related genes expression and signaling pathways were further confirmed in bulk RNA Sequencing data. Thus, this single-cell study will contribute novel insight into cellular landscape for deciphering intra-tumoral heterogeneity and understanding the molecular mechanism in tumor metastasis, with valuable significance for therapeutic management of PDAC.

## Results

### Single-cell transcriptome atlas in PDAC metastatic lesions

**Fig. 1:**
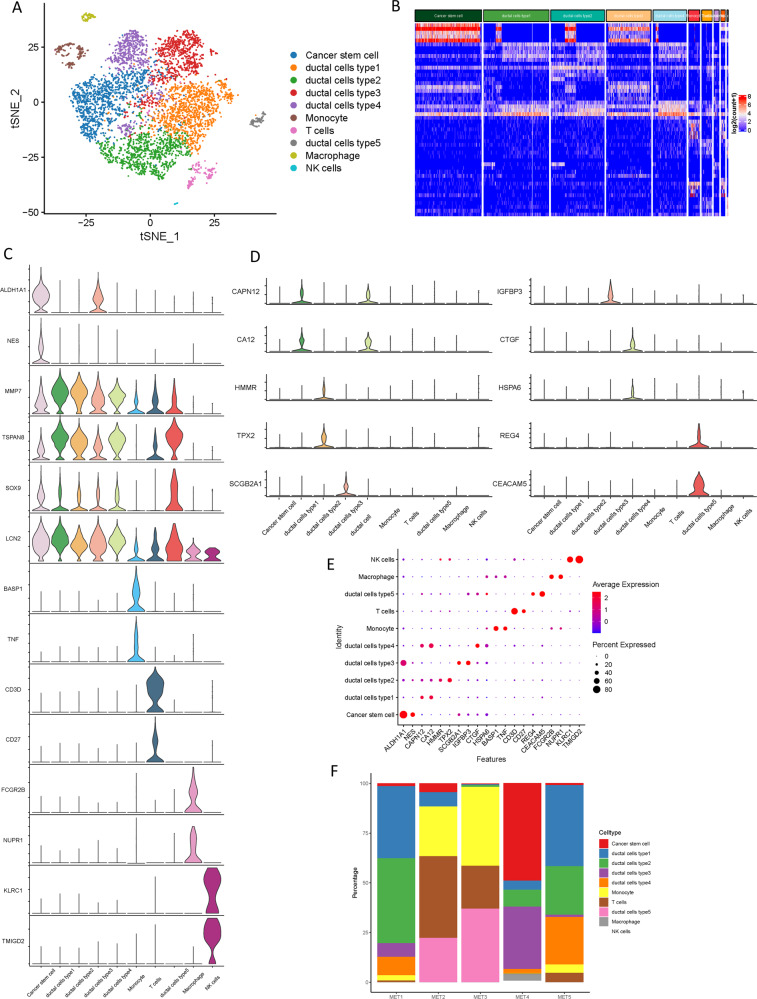
Single-cell transcriptomic analysis of metastatic PDAC lesions. **A** The t-distributed stochastic neighbor embedding (t-SNE) plot of the 10 identified main cell types in metastatic PDAC lesions. **B** Heatmap of top 5 DEGs among the myeloid cell subtypes, where the colors from red to blue represented alterations from high expression to low expression. **C** Violin plots showing the normalized expression levels of 14 representative canonical marker genes across the 10 clusters. **D** Violin plots showing the expression level of representative novel identified markers across the main cell types. **E** Dot plots showing the 20 signature gene expressions across the 10 cellular clusters. The size of dots represents the proportion of cells expressing the particular marker, and the spectrum of color indicates the mean expression levels of the markers (log1p transformed). **F** Relative proportion of each cell cluster across 5 metastatic PDAC lesions as indicated.

To investigate the cellular diversity in PDAC metastatic lesions, scRNA-seq analysis was implemented on metastatic tumor samples from 5 PDAC patients. After carrying out the initial quality control assessment described previously (Supplementary Fig. 1A and B), a total of 6,236 cells were profiled for subsequent analysis, of which single-cell transcriptomes were obtained. Following gene expression normalization, principal component analysis was performed on variably expressed genes (Supplementary Fig. 1C) across all cell samples. By using unbiased clustering based on t-distributed stochastic neighbor embedding (t-SNE) analyses, 10 main segregated cell clusters were identified in parallel (Supplementary Fig. 2A, Supplementary Table 1). Next, representative genes were developed by employing differential gene expression analysis to identify of each cell cluster according to expression profiles and well-known cell markers (Fig. 1A). And the clustering distribution of single cell from five patients is presented in Supplementary Fig. 2B and 2C. The top 5 differentially expressed genes (DEGs) for the cellular subclusters were presented in the heatmap (Fig. 1B), with more details extended in the following sections.

In particular, these cell lineages were as follows: (1) the Cancer stem cells characterized with high ALDH1A1 [8] and NES [9] expression; (2) the Ductal cells specifically expressing cell markers MMP7, TSPAN8, SOX9, and LCN2; [10, 11] (3) the Monocyte highly expressing BASP1 [12] and TNF; [13] (4) the T cells with high expression of CD3D [14] and CD27; [15] (5) the Macrophage specifically express the markers HLA − DRB5 and NUPR1; [16] (6) the NK cells expressing KLRC1 [17] and TMIGD2 [12]. The expression patterns of the cluster-specific marker genes in the cellular populations were demonstrated (Fig. 1C, Supplementary Fig. 3A). Notably, five distinct types of ductal cells were identified. Both types of ductal cells showed a high level of ductal markers: MMP7, TSPAN8, SOX9, and LCN2 [10, 11]. To characterize these ductal clusters, the expression profiles of subtype-specific marker genes were examined respectively (Fig. 1D). Results showed that CAPN12 and CA12 were highly expressed in type 1 ductal cells compared with other subtypes, suggesting suitable markers of CAPN12 and CAP12 for type 1 ductal cells. Type 2 ductal cells exhibited the highest expression levels of HMMR and TPX2, indicating that HMMR and TPX2 may identify these cells. SCGB2A1 and IGFBP3 were uniquely expressed in type 3 ductal cells, prompting reliable markers of SCGB2A1 and IGFBP3 for type 3 ductal cells. CTGF and HSPA6 were specifically expressed in type 4 ductal cells, thus, they could be used as specific markers for the detection of these cells. Type 5 ductal cells experienced unique expression of REG4 and CEACAM5, supporting specific markers of REG4 and CEACAM5 for type 5 ductal cells. Moreover, all ductal cells exhibited significant higher expression of reported poor prognosis and distant metastasis PDAC markers, such as CEACAM1/5/6 [18] and KRT19 [19], suggesting that these ductal subtypes might be malignant ductal cells (Supplementary Fig. 3B and C).

The dot plots illustrated the proportion of cells expressing representative markers and their scaled relative expression values in different cell clusters (Fig. 1E). The proportions of the cellular subpopulations diverse well among the tumor lesions (Fig. 1F), suggesting the intertumoral heterogeneity as well as the consistency among the lesions.

### Copy-number alterations in PDACs

**Fig. 2:**
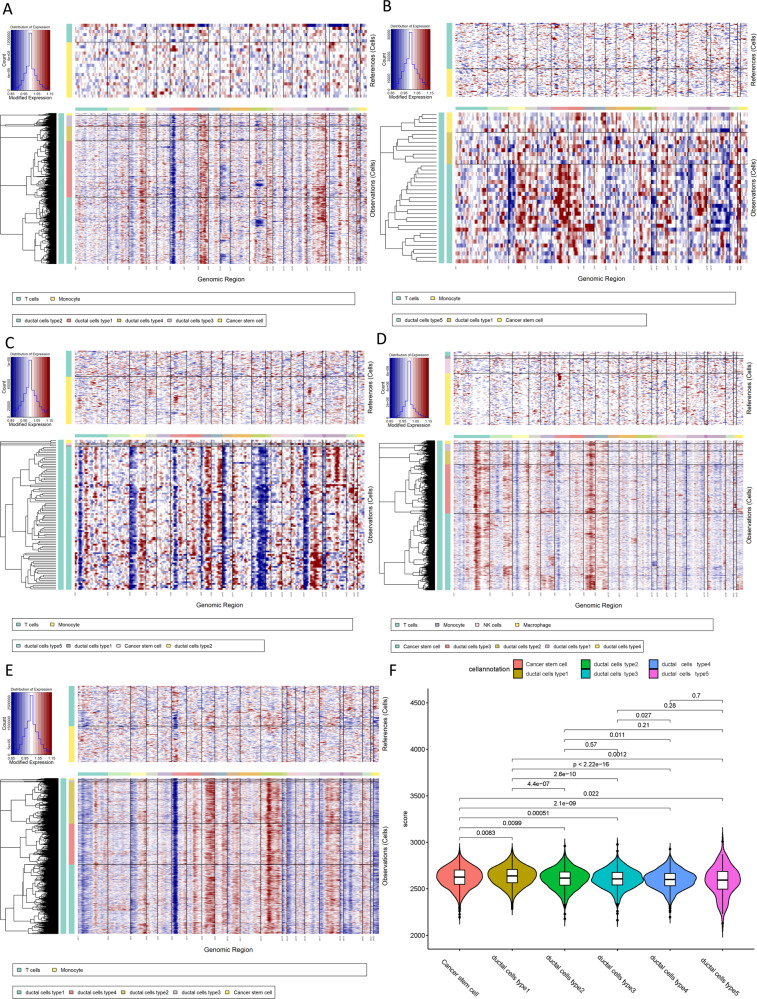
Copy-number variation analysis of PDAC cells. **A** The hierarchical heatmap showing large-scale CNVs in tumor lesions form one metastatic PDAC sample (MET1). **B** The hierarchical heatmap showing large-scale CNVs in tumor lesions form one metastatic PDAC sample (MET2). **C** The hierarchical heatmap showing large-scale CNVs in tumor lesions form one metastatic PDAC sample (MET3). **D** The hierarchical heatmap showing large-scale CNVs in tumor lesions form one metastatic PDAC sample (MET4). **E** The hierarchical heatmap showing large-scale CNVs in tumor lesions form one metastatic PDAC sample (MET5). **F** Violin plots showing distributions of CNV scores among different cell types.

To distinguish malignant cells, we calculated and identified large-scale chromosomal copy number variation (CNV) by inferCNV for each sample based on transcriptomes [20]. An inferCNV clustered heatmap was created per sample, which corresponds to the normalized expression values of immune cells plotted in the top panel and ductal cells in the bottom panel. In the resultant CNA heatmap, the regions of gain are depicted in red and regions of loss in blue. Most non-stromal cells presented relatively low CNV levels except for monocyte and T cells, which showed moderate CNV levels. We discovered that these five ductal cell subtypes experienced remarkable CNV events compared with cells in control samples (Fig. 2A–E). Notably, type 1 ductal cells presented significantly higher CNV levels than other types of ductal cells (Fig. 2F). These results suggested that five subtypes of ductal cells are all malignant cells in metastatic lesions.

### Transcriptional heterogeneity of malignant stromal cells

**Fig. 3:**
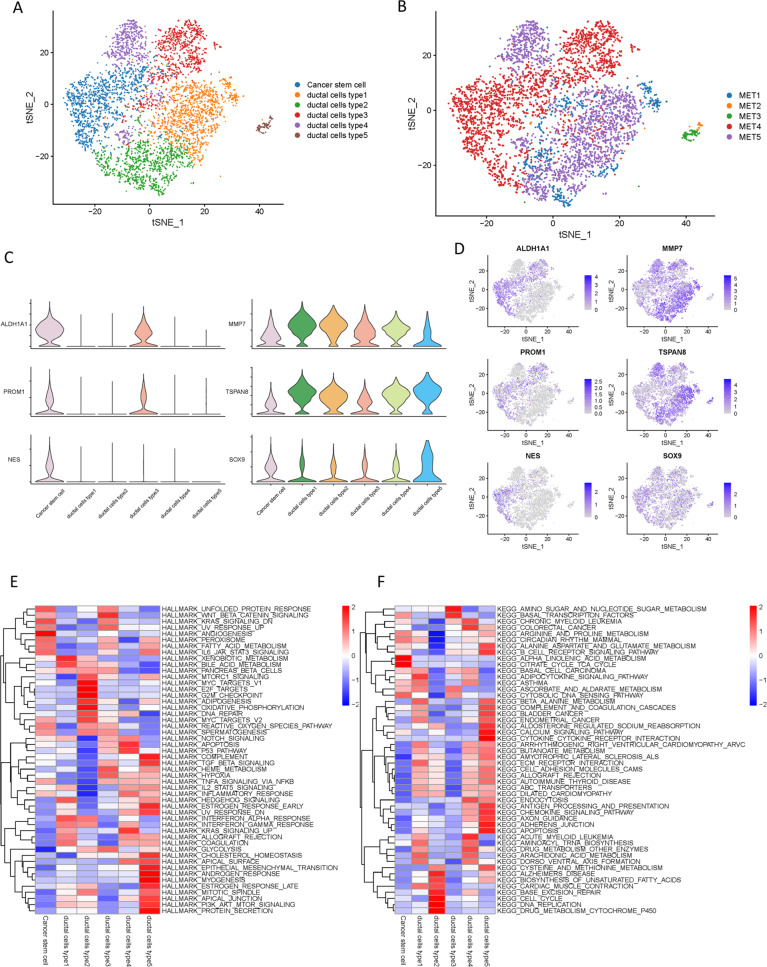
Distinct clusters of malignant cells in metastatic PDAC lesions. **A** t-SNE plot showing the 6 malignant cell subclusters from 5 PDAC samples. **B** Six malignant PDAC cell subclusters were identified by t-SNE analysis. **C** Violin plots for marker genes of cancer stem cells (ALDH1A1, PROM1, and NES) and ductal cells (MMP7, TSPAN8, and SOX9). **D** Feature plots for marker genes of cancer stem cells (ALDH1A1, PROM1, and NES) and ductal cells (MMP7, TSPAN8, and SOX9). The color legend shows the log1p normalized expression levels of the genes. **E** Heatmap showing the representative pathway terms of Hallmark enriched in each cellular subgroup. **F** Heatmap showing the representative pathway terms of KEGG enriched in each cellular subgroup.

Through the t-SNE analysis on malignant stromal cells, six cellular clusters in total, of which five belonged to ductal lineage and 1 was of the cancer stem lineage (Fig. 3A). The distribution of each cluster of single cells from five different patients was shown (Fig. 3B). The expression profiling in distinct subclusters of the malignant ductal and cancer stem cells were presented (Fig. 3C). As demonstrated in the top DEGs, specifically cancer stem lineage clusters expressed high levels of stemness feature genes including ALDH1A1, PROM1, and NES, while ductal lineage cluster expressed high levels of ductal markers such as MMP7, TSPAN8, and SOX9 (Fig. 3D).

To further reveal the biological roles of each cellular cluster in tumorigenicity and progression, competitive gene set variation analysis (GSVA) was performed (Fig. 3E, F). Type 1 ductal cells showed heightened activities of heterologous metabolism and bile acid metabolism processes and adipocytokine signaling pathway, indicating that type 1 ductal cells were identified as cells of relatively low malignancy. Most genes with high expression levels in type 2 ductal cells were enriched in cell cycle and DNA replication, suggesting that type 2 ductal cells were associated to tumor proliferation and growth. Upregulated genes expressed in type 3 ductal cells were mainly mediated in tumor migration-related processes like Wnt/β-catenin and KRAS signaling pathways, indicating that type 3 ductal cells were identified as cells of relatively high malignancy. Type 4 ductal cells experienced high enrichment involved in tumor progressions, such as p53, KRAS, and apoptosis-related signaling pathways, highlighting that type 3 ductal cells were also malignant cells. Notably, type 5 ductal cells mainly enriched with IL6/JAK/STAT3, PI3K/AKT/MTOR and TGF-β signaling pathways and Epithelial-Mesenchymal Transformation, supporting that type 5 ductal cells were the major factor of malignant cells in metastatic lesions.

### Gene expression patterns in ductal cells during PDAC progression

**Fig. 4:**
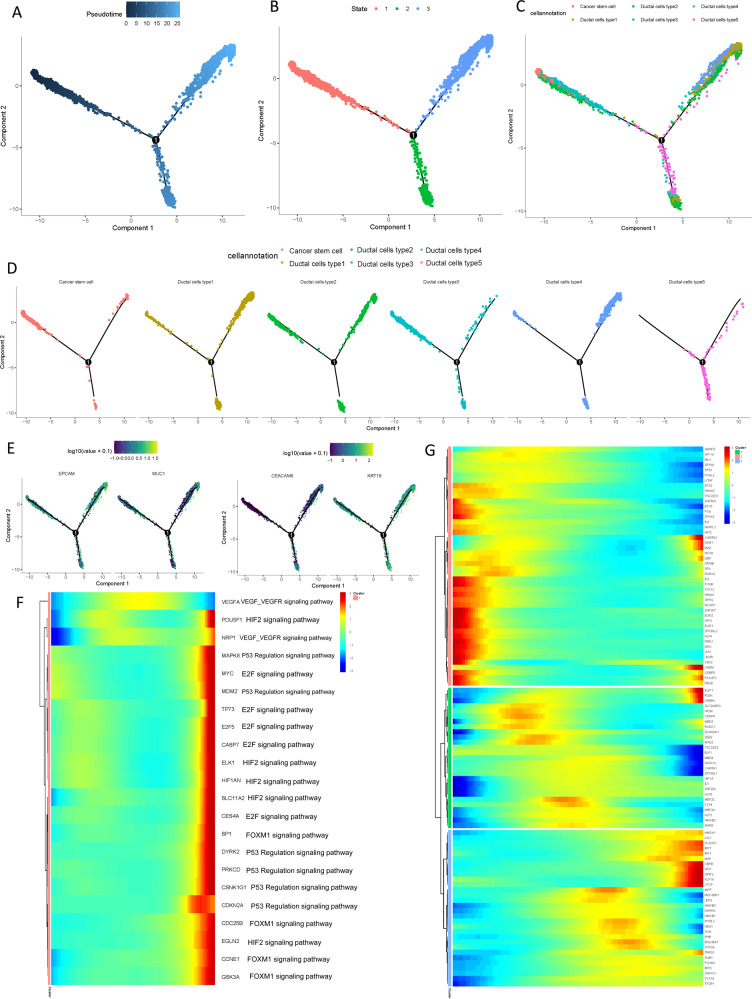
Simulation of the development trajectory of malignant cells and the analysis of gene expression pattern inferred by Monocle2. Simulation of the differentiation trajectory of malignant cells group from PDAC, **A** pseudo-trajectory of malignant cells, **B** cell type transition, **C** cell source transition. **D** Pseudotime trajectory of malignant cells from different cells. Each point corresponds to a single cell. **E** Expression of representative genes (EPCAM, KRT19, MUC1, and CEACAM6) are mapped to the single-cell trajectory plot. Color key from gray to red indicates relative expression levels from low to high. **F** Heatmap showing expression of representative known PDAC associated genes across single cells. Corresponding pathways for each gene were also shown. Color key from blue to red indicates relative expression levels from low to high. **G** Heatmap showing expression of representative identified TFs across single cells. Color key from blue to red indicates relative expression levels from low to high.

There is not a consensus yet regarding which pancreatic cell type being responsible for the point of origin of tumor cells. To further elucidate the origins of neoplastic cells in the carcinogenesis of PDAC, pseudotime trajectory analysis was performed using cancer stem cells and five types of ductal cells. R package monocle was then used to sort individual cells by these genes and construct the tree-like structure of the entire lineage differentiation trajectory (Fig. 4A). Along with trajectory progression, cells experienced three states: starting point of branching (pre-branch) and the two other branches (Fate1 and Fate2; Fig. 4B). Cancer stem cells and type 3 ductal cells were mainly shown in the beginning of the trajectory. Type 1 and 4 ductal cells predominately appeared at the end of the trajectory branch 1 and type 5 ductal cells were mostly at the end of the trajectory branch 2 (Fig. 4C, D). Notably, type 2 ductal cells were appeared at the ends of different branches, suggesting transitional form of type 2 ductal cells for tumor progression. Our findings indicated the possibility that both cancer stem cells and type 3 ductal cells could be the origin of malignant cells and may transform to two distinct types of neoplastic ductal cells during the PDAC progression. Along with the trajectory, epithelial cell marker EPCAM and ductal marker KRT19 exhibited sustained high expression levels during PDAC progression, while genes previously reported to be involved in tumor progression, such as MUC1 and CEACAM6, gradually upregulated along with the transition (Fig. 4E).

Moreover, the gene expression patterns mediated in the cell state transition along with progression trajectory were dissected and 7603 dynamical genes with significant expression changes were determined (Supplementary Table 2). Particularly, the alteration of multiple crucial drivers participated in the PDACs cacogenesis (Fig. 4F), including several regulators potentially mediated in progression of PDAC, such as VEGF/VEGFR, HIF2, and P53 signaling pathway. Meanwhile, the transcriptional factors involved in immune cell differentiation and proliferation, such as MEF2C, HMGB1, HMGB2, CREM, LITAF, ID1, and ID3, etc. (Fig. 4G), were remarkably dysregulated with the trajectory transition process.

**Fig. 5:**
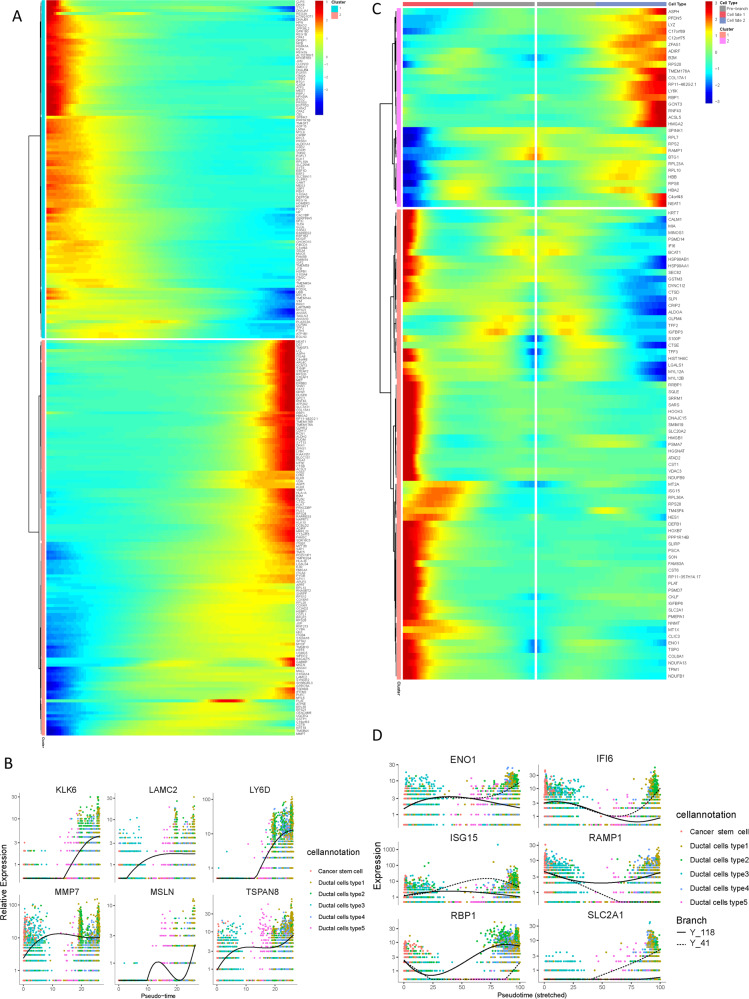
Differential gene expression profiles along malignant progression. **A** Heatmap hierarchical clustering showing differentially expressed transcription factor genes along with the pseudotime curve. Color key from blue to red indicates relative expression levels from low to high. **B** Expression patterns of representative differentially expressed genes in the progression process. **C** The pseudotime heatmap displayed the differentially expressed genes between the branches according to the BEAM analysis. Color key from blue to red indicates relative expression levels from low to high. **D** Expression patterns of representative differentially expressed genes between different branches in the progression process.

Subsequently, these genes were further subclustered into initial expression patterns and terminal patterns with specific expression patterns (Fig. 5A). In addition, the potential key genes associated with distant metastasis of PDAC, such as MMP7, TSPAN8, MSLN, LAMC2, KLK6, and LY6D (Fig. 5B), were discovered to significantly change along with progression trajectory. Next, branched heatmap was employed to present the gene pattern of distinct cell fate branches, genes are assigned into two different clusters based on the expression dynamics (Fig. 5C; Supplementary Table 3). Genes most significantly diverted between two branches were recognized: Alpha-enolase (ENO1), Interferon-α inducible protein 6 (IFI6), Interferon-stimulated gene product 15 (ISG15), receptor activity modifying protein 1 (RAMP1), Retinoblastoma binding protein-1 (RBP1), and specific glucose transporter GLUT1 (SLC2A1; Fig. 5D).

### Complex cell–cell communication networks in the TME

**Fig. 6:**
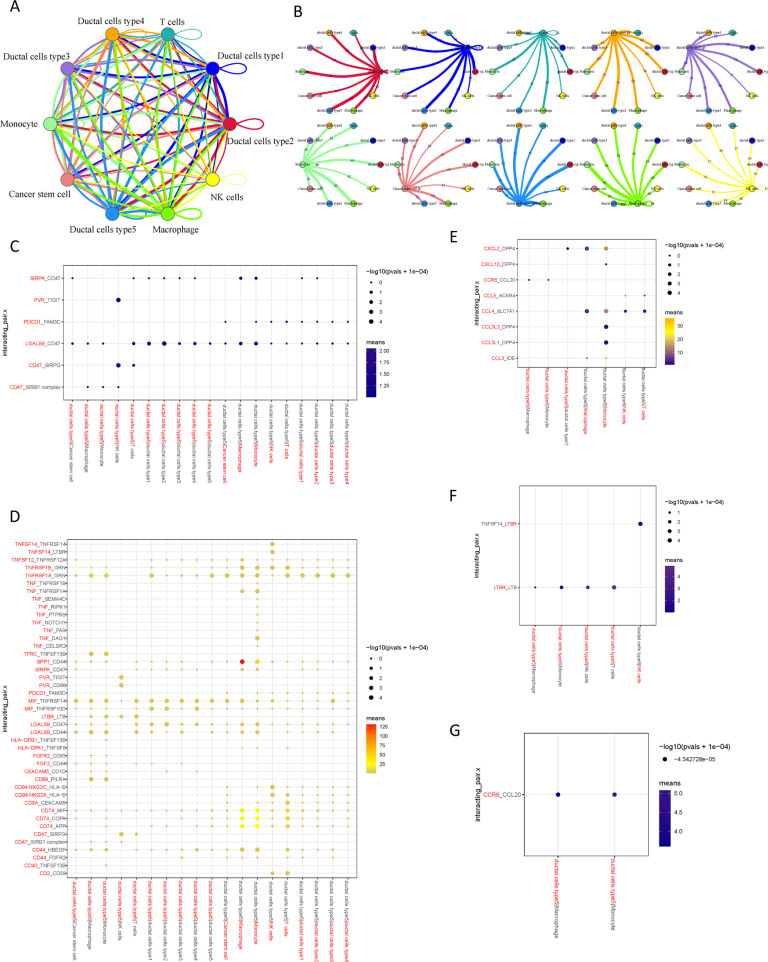
The dense network and multiple regulatory immune responses in the TME. **A** Capacity for intercellular communication between malignant cells and immune cells. Each line color indicates the ligands expressed by the cell population represented in the same color (labeled). The lines connect to the cell types that express the cognate receptors. The line thickness is proportional to the number of ligands when cognate receptors are present in the recipient cell type. The loops indicate autocrine circuits. The map quantifies potential communication but does not account for the anatomical locations or boundaries of the cell types. **B** Detailed view of the ligands expressed by each major cell type and the cells expressing the cognate receptors primed to receive the signal. Numbers indicate the quantity of ligand–receptor pairs for each intercellular link. Overview of selected ligand–receptor interactions of type 5 ductal cells under inhibitory interaction (**C**), stimulatory interaction (**D**), chemokines interaction (**E**), Th1-mediated interaction (**F**), and Th17-mediated interaction (**G**). *P* values are indicated by circle size, with the scale to the right (permutation test). The means of the average expression levels of interacting molecule 1 in cluster 1 and interacting molecule 2 in cluster 2 are indicated by color. Assays were carried out at the mRNA level but were used to extrapolate protein interactions.

To characterize the tumor microenvironment of PDAC metastatic lesion, CellPhoneDB was applied to detected the intercellular communication among malignant cell types and immune cells. In the output of the results of the ligand receptor, the heatmap plot function is used to analyze the interaction between immune cells. The results showed that macrophages and monocytes were significantly active and interacted with a variety of cells (Supplementary Fig. 4A and B). Broadcast ligand–receptor pairs were identified demonstrated extensive molecular interactions among the major cell types (Fig. 6A, B).

Previous results suggested that type 5 ductal cells may be the main source of malignant cells, thus, the ligand-receptor complex among type 5 ductal cells and other major cell types were investigated. Notably, inhibitory receptor–ligand pairs between type 5 ductal cells and immune cells were widely identified. In addition to CD47-SIRPG and CD47-SIRB1 complex, other cell–cell interactions that have rarely been reported in PDAC (e.g., PVR-TIGIT, PDCD1-FAM3C, and LGALS9-CD47) were also observed (Fig. 6C). Interestingly, costimulatory interactions between type 5 ductal cells and immune cells were also widely discovered (Fig. 6D), from which specific ligand–receptor complexes (e.g., TNFRSF1A-GRN, MIF-TNFRSF14 and LGALS9-CD44) were identified. Of note, macrophage expressed relatively high levels of SPP1, and the ligands of SPP1 (CD44) were overexpressed by type 5 ductal cells, indicating the presence of functional interactions between macrophage and type 5 ductal cells. Macrophage participated in the pancreatic cancer metastasis, which was demonstrated by previous reports [21]. In addition, chemokines were relatively higher expressed in immune cells (e.g., CCL4, and CXCL2), while the type 5 ductal cells commonly expressed high levels of the corresponding receptors, indicating that these chemokines served as critical players in regulating diverse types of immune cells infiltration in PDAC tumor microenvironment (Fig. 6E). The previously reported T helper (Th) 1 immune response could be used as a biological marker for immunotherapy in metastatic lymph nodes of PDAC [22]. Specifically, the interactions of Th1-mediated responses between immune cells and type 5 ductal cells (Fig. 6F) suggested that LTBR and its ligands may be the crucial drivers in aggressiveness of PDAC. Moreover, the putative ligands for which cognate receptors of Th17-mediated responses were also identified, such as the intercellular communications produced by CCR6-CCL20 (Fig. 6G). corresponding to the recent study supporting pathogenic TH17 responses are be demonstrated to be responsible for neoantigen-induced tumor progression in PDAC [7].

### Mapping malignancy-specific regulon networks by SCENIC

In addition, single-cell regulatory network inference and clustering (SCENIC) was employed to further explore the difference of potential regulons (i.e., transcriptional factors (TFs) and their target genes) activity of between type 5 ductal cell-specific and other cellular clusters.

**Fig. 7:**
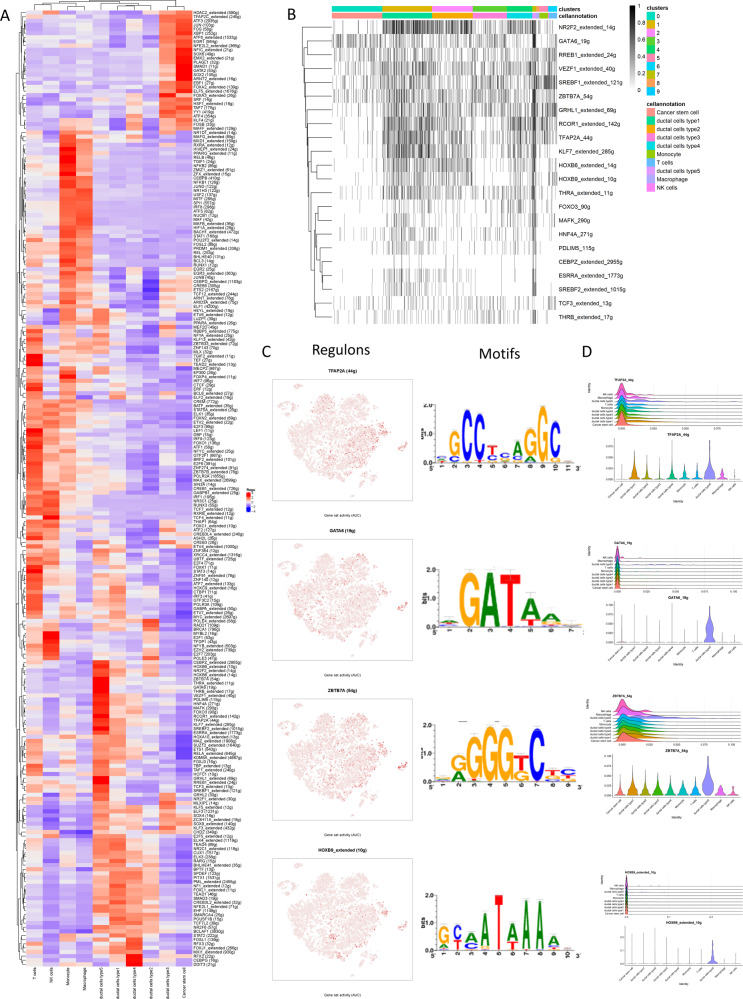
Specific transcription factors involved in malignant progression. **A** The heatmap exhibits the average regulon activities of transcription factors in different cellular types, color key from blue to red indicates relative expression levels from low to high. **B** Heatmap of regulon activity analyzed by SCENIC with default thresholds for binarization. The “regulon” refers to regulatory network of TFs and their target genes. “On” indicates active regulons; “Off” indicates inactive regulons. **C** The t-SNE plot shows the top four highly expressed transcription factors in type 5 ductal cells, and part of their motifs were listed in the right panel. **D** Violin plots showing distributions of representative TFs expression among different cell types.

Based on 337 regulons activity with 35,684 filtered genes with default filter parameters (Supplementary Fig. 5A), the regulon activity could also distinguish subclones of the different ductal subtypes (Fig. 7A). Accordingly, type 5 ductal cell-specific regulon activity was binarized and matched with cellular clusters (Fig. 7B). Next, TFAP2A, GATA6, ZBTB7A, and HOXB9 were identified as candidate TFs with specifically regulated expressions in type 5 ductal cell clusters, in addition, corresponding motifs were listed (Fig. 7C). The expression patterns of the representative TFs in the cellular populations were demonstrated (Fig. 7D and Supplementary Fig. 5B–E).

### Validation of metastatic-related hub genes and signaling pathways in bulk RNA-sequencing data

**Fig. 8:**
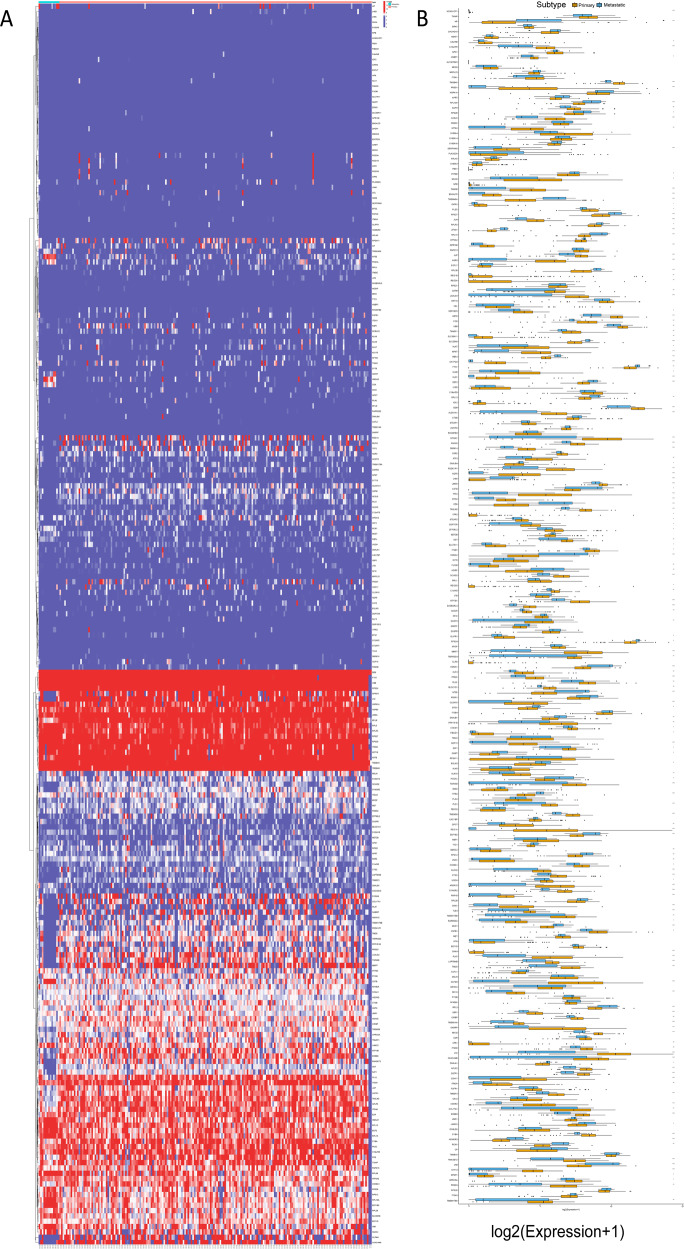
Expression profiling of metastatic-related genes in primary/metastatic tumor samples. **A** Heatmap of metastatic-related genes was drawn to reveal different distribution of expression states, where the colors of red to blue represented alterations from high expression to low expression. **B** Comparison of the expression level of metastatic-related genes between primary and metastatic cancer samples.

**Fig. 9:**
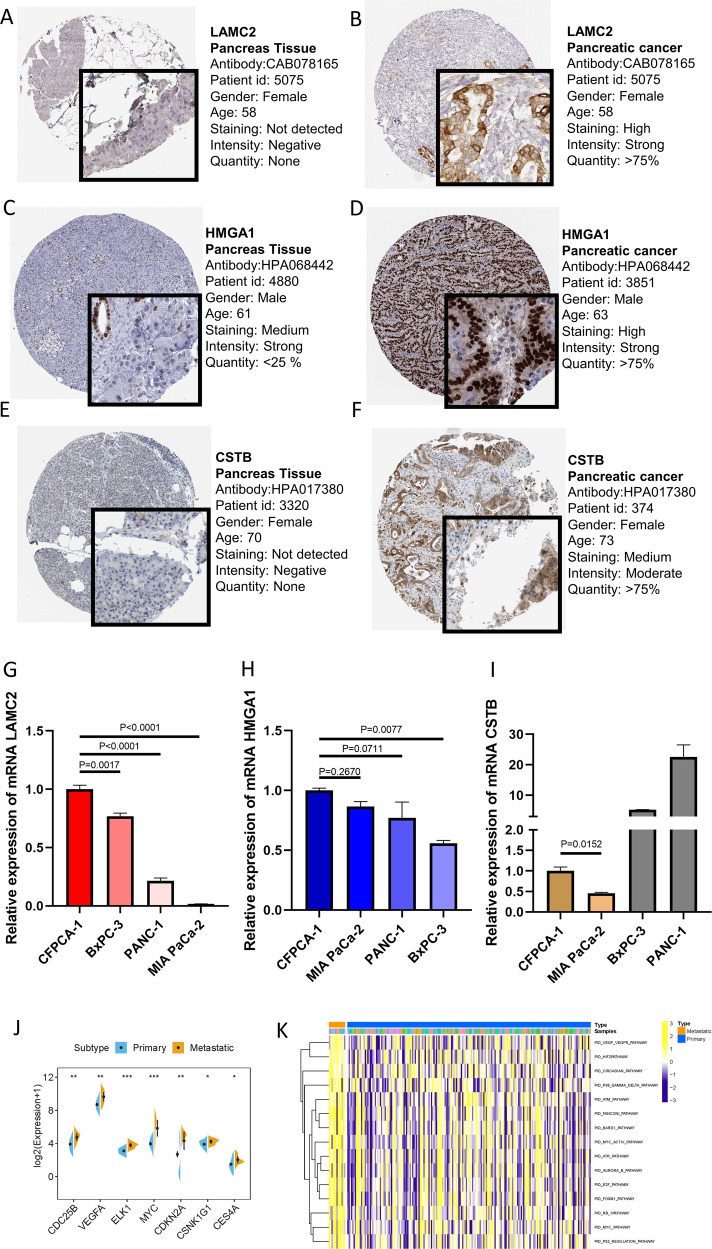
Validation of expression patterns of representative metastatic-related genes. Differentially expressed proteins of LAMC2 in normal (**A**) and pancreatic cancer tissues (**B**) in the Human Protein Atlas database. Differentially expressed proteins of HMGA1 in normal (**C**) and pancreatic cancer tissues (**D**) in the Human Protein Atlas database. Differentially expressed proteins of CSTB in normal (**E**) and pancreatic cancer tissues (**F**) in the Human Protein Atlas database. **G** LAMC2 were overexpressed in metastatic cell lines relative to primary cell lines. **H** HMGA1were overexpressed in metastatic cell lines relative to primary cell lines. **I** CSTB were overexpressed in metastatic cell lines relative to primary cell lines. Enrichment of metastatic-related signaling pathways in primary/metastatic tumor samples. **J** Relative expression levels of hub genes in metastatic-related signaling pathways were upregulated in metastatic lesions compared with primary samples. **K** GSVA enrichment analysis showing the activation states of biological pathways in primary/metastatic PDAC samples. The heatmap was used to visualize these biological processes, and yellow represented activated pathways and blue represented inhibited pathways.

To validate the metastasis-associated genes and signaling pathways discovered in the aforementioned results, PDAC cohort (ICGC-PACA-AC) were used in the subsequent analyses. Firstly, expression distribution of metastasis-related genes in primary and metastatic tumor samples was plotted (Fig. 8A). Then, expression levels of metastasis-related genes were compared between primary tumors and metastatic lesions. As shown in Fig. 8B, most metastasis-related genes experienced significantly dysregulation in metastatic tumors, consistent with previous findings. The HPA database was used to explore protein expression levels in PDAC samples. The results showed that relative to normal samples, proteins (LAMC2, HMGA1, CSTB) were significantly upregulated in tumor tissues (Fig. 9A–F). In addition, their mRNA expression levels were also investigated in pancreatic cancer cell lines (Fig. 9G, H and I). Our results indicated that LAMC2, HMGA1, and CSTB showed significantly higher trends in CFPAC-1 cell lines.

Moreover, representative hub genes expression levels were significantly elevated in metastatic lesions than primary samples (Fig. 9J). Moreover, GSVA analysis between primary and metastatic tumors was performed (Fig. 9K). The results of GSVA showed that enrichment pathways of metastatic lesions prominently associated with tumorigenesis-relevant processes, including VEGF/VEGFR, HIF2, E2F, FOXM1, and P53 regulation pathways, which was in accordance with the previous results (Fig. 4F).

## Discussion

PDAC was considered as malignant tumor with high intra-tumoral heterogeneity and aggressive features which brings great challenges to both early precision diagnosis and effective personalized treatment. Therefore, there was an urgent need to reveal the underlying molecular mechanism of intra-tumoral heterogeneity and tumor metastasis characteristics with high cellular resolution in the TME of metastatic PDAC.

Herein, 10 predominant populations of cells with t-SNE clustering were identified in the metastatic PDAC biopsies that included 6 stromal cellular types and 4 immune cell clusters. Their molecular and cellular features were also characterized with regard to their role in the tumorigenesis of PDAC. Notably, five types of ductal cells with distinct transcriptomic patterns, were recognized. According to subsequent CNV level analysis, all five types ductal cells presented in metastatic lesions were demonstrated to be malignant cells and type 1 ductal cells experienced the highest CNV scores. GSVA on each subgroup of malignant cells demonstrated the heterogeneity of underlying biological mechanism in distinct types of cells. Interestingly, the unique functions of type 5 ductal cells were significantly related to Epithelial-Mesenchymal Transformation (EMT), which contributed into the capability to invade surrounding tissues and travel through the peripheral circulation. Since EMT+ cancer cell subpopulations were correlated with poorer patient prognosis and more aggressive disease [23], our findings suggested pivotal roles of type 5 ductal cells for distant metastasis in PDAC.

In addition, the activation EMT- and TGF-β-related pathways participated in the formation of peritumoral stroma [24]. Targetable TGF-β molecular blockaders have been demonstrated to reprogram the contexture of TME and reshape the anti-cancer immunology [25, 26]. Thus, it recommended that the type 5 ductal cellular populations might be suitable for the administration of TGF-β inhibitors.

It is well-established that cancer stem cells as the main drivers of tumorigenicity were the origins of neoplastic cells in PDAC [27]. However, mounting studies demonstrated the origin of malignant cells derived from ductal cells [28, 29]. Herein, pseudotime trajectory analysis using Monocle also indicated that tumor cells may derive from both cancer stem cells and type 3 ductal cells transdifferentiation.

Notably, various canonical oncogenic pathways, such as VEGF/VEGFR [30], HIF2 [31], E2F [32], FOXM1 [33], and P53 regulation pathways [34], were activated during trajectory progression. Moreover, these results were validated between primary and metastatic cancer samples in bulk RNA sequencing data. Our findings offer novel insight to support that these signaling pathways could potentially serve as underlying molecular mechanism of metastasis in PDAC. Nonetheless, our findings should be confirmed in future studies. In addition, LAMC2, HMGA1 and CSTB were discovered to be upregulated significantly along with trajectory transition and elevated in the metastatic tumor lesions, which was consistent with published researches focusing on biological roles of LAMC2/ HMGA1 [35, 36]. However, it was little to known the CSTB-mediated underlying mechanism in invasiveness of PDAC and CSTB-targeting precision interventions might represent promising therapeutic strategies, which required further investigation in future studies. Notably, the expression level of MET was increased during tumor progression. This finding is consistent with metastasis theory in which EMT-tumor cells regained its epithelial ability to form metastatic sites, suggesting the metastatic lesions were originated from cancer cells that have undergone EMT [37]. We also discovered some unidentified regulators such as TM9SF3, LYZ, and ARL4C, which are potentially engaged in the cellular transition from the cancer stem cells into metastatic malignant cells. The above results indicate that both cancer stem cell as well as type 3 ductal cells might be the origin of pancreatic neoplastic cells lineages, which facilitated enhancing our understanding of PDAC carcinogenesis.

Moreover, the intimate cell–cell communication among type5 ductal cell and major cell types were identified in the TME. High expression of inhibitory receptor–ligand complex, like CD47-SIRPG, may be crucial role of immunotherapeutic resistance in PDAC. Moreover, upregulated activity of chemokine, such as CCL4, and CXCL2, between tumor cancer and immune might be the hub driver of recruitment of infiltrating immune cells. Macrophage with high expression of costimulatory ligands exhibited the most complex interaction with other cell types, indicating potential anti-cancer effect of these macrophages in PDAC.

Meanwhile, a network of regulons was established using the SCENIC analysis, that can be identified as potential candidates for type 5 ductal cell-specific TF programs. By employing this algorithm, several specific TFs in type 5 ductal cells were determined, such as TFAP2A, GATA6, HOXB9, and ZBTB7A. Overexpression of TFAP2C was demonstrated to resensitize tumor cells to gemcitabine in PDAC [38]. The biological function of GATA6 in PDAC remains controversial yet. A previous study reported that GATA6 inhibits the epithelial-mesenchymal transition (EMT) in vitro, however, high GATA6 levels are related with better prognosis and are found in well-differentiated tumors [39]. Downregulation of HOXB9 mediated by TGF-β1-induced Kindlin-2 expression was correlated with PDAC progression [40]. However, the potential role of ZBTB7A is still elusive in PDAC, which needs further exploration.

## Conclusions

In our work, the intratumoral heterogeneity of PDAC cells were demonstrated with corresponding molecular characteristics and transcriptome features identified. In addition, the cellular lineage transition of malignant cells from original cells in metastatic lesions. Moreover, complex interactions of major cell types were deciphered in the TIME of PDAC. In addition, regulons networks were established to determine the candidates of cell type-specific transcriptional factors (TFs) and their target genes. Moreover, metastatic-related genes expression and signaling pathways activity were further confirmed in bulk RNA Sequencing data. In conclusion, this study lays a new foundation for the identification of therapeutic targets to enhance precision therapeutic strategy in metastatic PDAC.

## Materials and methods

### Sources of datasets

The single-cell RNA sequencing information of GSE154778 were obtained from Gene Expression Omnibus (GEO) database, which contained 6,236 cell samples from 5 liver metastatic PDAC biopsies. The scRNA-seq gene-barcode matrix, features data and UMI count tables of barcodes has been described by Lin et al. [41]. The RNA sequencing profile of the patients from ICGC-PACA-CA dataset, which contains 195 primary tumor samples and 13 metastatic cancer lesions, was obtained from ICGC portal (https://dcc.icgc.org/). There was no necessity to obtain Ethics Committee approval, owing to all information were publicly available and open-access. The Human Protein Atlas (http://www.proteinatlas.org) was used to investigate the protein levels of metastatic-related genes.

### Quality control and the dimensionality reduction

The Seurat object with gene expression data was imported into the Seurat (v2.3.0) R toolkit with the Read10× () function [42]. Gene-cell matrixes were filtered to exclude cells (<500 transcripts/cell, >5% mitochondrial genes) and genes (<1000 cells/gene and >200,00 cells/gene). For each sample, the gene expression was represented as the fraction of the gene and multiplied by 10,000, which were converted into natural logarithm and normalized after adding 1 to avoid taking the log of 0. The top 1000 highly variable genes (HVGs) from the normalized expression matrix were generated to perform the principal component analysis (PCA) based on these HVGs. Significant principal components were determined using Jackstraw analysis and visualization of heatmaps focusing on PCs 1 to 40. PCs 1 to 13 were employed for graph-based clustering (at res = 0.5) to identify distinct groups of cells. The following Seurat functions (FindNeighbors and FindClusters) were used to calculate the dimension-reduction coordinates. Finally, single-cell clustering was visualized by t-SNE (t-Distributed Stochastic Neighbor Embedding).

### Cell-clustering and annotation

The cluster-specific marker genes were identified by running the FindAllMarkers function in the Seurat package using the default non-parametric Wilcoxon rank sum test with Bonferroni correction. To identify differentially expressed genes between two clusters, we used the ‘find.markers’ function. The cell groups were annotated based on the DEGs and the well-known cellular markers from the literature. Detailed information of the cell markers was recorded in Supplementary Table 1.

### Single-cell copy-number variation (CNV) evaluation

The CNV evaluation of each cell was conducted by infercnv R package (version 1.4.0; https://github.com/broadinstitute/inferCNV/wiki) [20]. The CNVs of Epithelial cells were calculated and the immune cells were applied as the reference. The inferCNV analysis was performed with parameters including “denoise”, default hidden Markov model (HMM) settings, and a value of 0.1 for “cutoff”. To reduce the false-positive CNV calls, the default Bayesian latent mixture model was implemented to identify the posterior probabilities of the CNV alterations in each cell with the default value of 0.5 as the threshold.

### DEGs identification and GSVA

We identified the differentially over-expressed genes in the specific cluster when compared to other remaining clusters with the Wilcoxon Rank-Sum Test with the FindMarkers function in Seurat (adjusted *P*-value < 0.05, only.pos = TRUE and logfc.threshold = 0.25). Predominantly, pathway analyses were carried out to evaluate activation of hallmark pathways and metabolic pathways, which were described in the MSigDB databases (https://www.gsea-msigdb.org/gsea/msigdb) [43]. Then, we applied GSVA [44] in the GSVA package (version 1.36.3) to assign pathway activity estimates to assess the relative pathway activities in the Cancer stem cells and epithelial cells.

### Constructing single-cell trajectories in PDAC

The R package Monocle2 (v2.16.0) was applied to conduct single-cell trajectory analysis with the assumption that one-dimensional ‘time’ can describe the high-dimensional expression values to discover the cell-state transitions [45]. The clusters identified as Cancer stem cell and Epithelial cells were loaded into R environment. The newCellDataSet function was applied to create an object with the parameter expressionFamily = negbinomial.size. In the trajectory analysis, we used genes meeting the thresholds that mean_expression ≥ 0.1 and dispersion_empirical ≥ 1 * dispersion_fit identified by Monocle2 to sort cells in pseudo-time order. The reduceDimension() function using the parameters reduction_method = “DDRTree” and max_components = 2 was applied to reduce dimensions and the visualization functions ‘plot_cell_trajectory’ were used to plot the minimum spanning tree on cells. Genes that changed along with the pseudotime were calculated (*q*-val < 10 − 5) by the “differentialGeneTest” function and visualized with the plot_pseudotime_heatmap and the genes were clustered into subgroups according to the gene expression patterns. Detailed information of the cell markers was recorded in Supplementary Table 2. To identify the genes that separate cells into branches, the branch expression analysis modeling (BEAM) analysis were performed and genes resulting from the BEAM analysis with a *q*-value < 10 − 10 were separated into groups and visualized with the plot_genes_branched_heatmap() function. Detailed information of the cell markers was recorded in Supplementary Table 3.

### Cell–cell communication analysis

To investigate potential interactions across different cell types in the TME, cell–cell communication analysis was performed using CellPhoneDB, which is a publicly available repository of curated receptors and ligands and their interactions [46]. CellPhoneDB analysis was performed using the CellPhoneDB Python package (2.1.7). Single-cell transcriptomic data of cells annotated as Cancer stem cells, ductal cells type 1-5, macrophages, T cells, NK cells and monocyte were input into CellPhoneDB for cell–cell interaction analysis. Enriched receptor–ligand interactions between two cell types were derived based on the expression of a receptor by one cell type and the expression of the corresponding ligand by another cell type. Then, we identified the most relevant cell type-specific interactions between ligands and receptors, and only receptors and ligands expressed in more than 10% of the cells in the corresponding subclusters were considered.

Pairwise comparisons were performed between the included cell types. We first randomly permuted the cluster labels of all cells 1000 times to determine the mean of the average receptor and ligand expression levels of the interacting clusters. This generated a null distribution for each receptor–ligand pair. By calculating the proportion of the means that were higher than the actual mean, a *P* value for the likelihood of the cell-type specificity of the corresponding receptor–ligand complex was obtained. We then selected interactions that were biologically relevant.

### The regulon activity of TFs with SCENIC

The SCENIC algorithm had been developed to assess the regulatory network analysis regard to TFs and discover regulons (that is, TFs and their target genes) in individual cells. Log-normalized expression matrix with gene names in rows and cells in columns generated using Seurat was input to SCENIC (version 1.2.4) [47]. Then, motif dataset (hg19-tss-centered-10kb-7species.mc9nr.feather) was used to construct regulons for each TF in SCENIC. The co-expressed genes for each TF were constructed with GENIE3 software, followed by Spearman’s correlation between the TF and the potential targets, and then the “runSCENIC” procedure assisted to generate the GRNs (also termed regulons). Finally, regulon activity was analyzed by AUCell (Area Under the Curve) software, where a default threshold was applied to binarize the specific regulons (“0” present “off” of TFs, and “1” refer to “on”). Detailed information of the cell markers was recorded in Supplementary Table 4.

### Experimental validation

CFPAC-1 (metastatic pancreatic cell line) and three primary pancreatic cancer cell lines (BxPC-3 cells, MiaPaCa-2 cells, and PANC-1 cells) were purchased from the Cell Bank of the Type Culture Collection of the Chinese Academy of Sciences, Shanghai Institute of Biochemistry and Cell Biology. All cells utilized were free of mycoplasma contamination and regularly tested for mycoplasma and purity of the culture by Short-tandem repeat polymorphism analysis (STR) profiling. The cell lines were all cultured in Roswell Park Memorial Institute (RPMI-1640) medium plus 10% fetal bovine serum (FBS; Invitrogen, Carlsbad, CA, USA). All cell lines were grown without antibiotics in a humidified atmosphere of 5% CO_2_ and 99% relative humidity at 37 °C. Three different cell lines were subjected to quantitative real-time polymerase chain reaction (qRT-PCR). Quantitative real-time PCR was analyzed as described previously [48]. All samples were analyzed in triplicates. Glyceraldehyde-3-phosphate dehydrogenase (GAPDH) levels were used as the endogenous control and relative expression of LAMC2/CSTB/HMGA1 was calculated using the 2-ΔΔCt method. The sequences of primers used for PCR were as follows: LAMC2, 5′- TCGGGATACTCACAGGCTCATCAC-3′ (forward) and 5′- GTGGTCTGAGGCAGGAATGTTAGTG-3′ (reverse); CSTB, 5′- GTGAGGTCCCAGCTTGAAGAGAAAG-3′ (forward) and 5′- GCGACCACCTGGCTCTTGAATG-3′ (reverse); HMGA1, 5′- CGAAGTGCCAACACCTAAGAGACC-3′ (forward) and 5′-GATGCCCTCCTCTTCCTCCTTCTC-3′ (reverse); and GAPDH, 5′- CAGGAGGCATTGCTGATGAT-3′ (forward) and 5′-GAAGGCTGGGGCTCATTT-3′ (reverse).

### Statistical analysis

All statistical analyses were performed using R (http://www.r-project.org). We do not display each data point in all box and violin plots because a large number of data points would obscure the overall distribution. A two-sided paired or unpaired Student’s *t*-test and unpaired Wilcoxon rank-sum test was used where indicated. *P* < 0.05 was considered to indicate statistical significance.

## Supplementary information

Supplementary Figures

Supplementary Tables

## Data Availability

The datasets generated for this study can be found in the GEO database (https://www.ncbi.nlm.nih.gov/geo/) and the ICGC dataset (https://dcc.icgc.org/). All the data generated or analyzed during this study are included in this article and its supplementary information files or available from the author upon reasonable request.

## Code availability

R and other custom scripts for analyzing data are available upon request.
