# Supplementary material for: Single-cell RNA transcriptome reveals the intra-tumoral heterogeneity and regulators underlying tumor progression in metastatic pancreatic ductal adenocarcinoma
Source: Cell Death Discov. 2021 Nov 3;7:331. doi: 10.1038/s41420-021-00663-1 (PMC8566471; doi:10.1038/s41420-021-00663-1)
Supplement: Supplementary file 1 — Supplementary Figures [file 41420_2021_663_MOESM1_ESM.pdf]

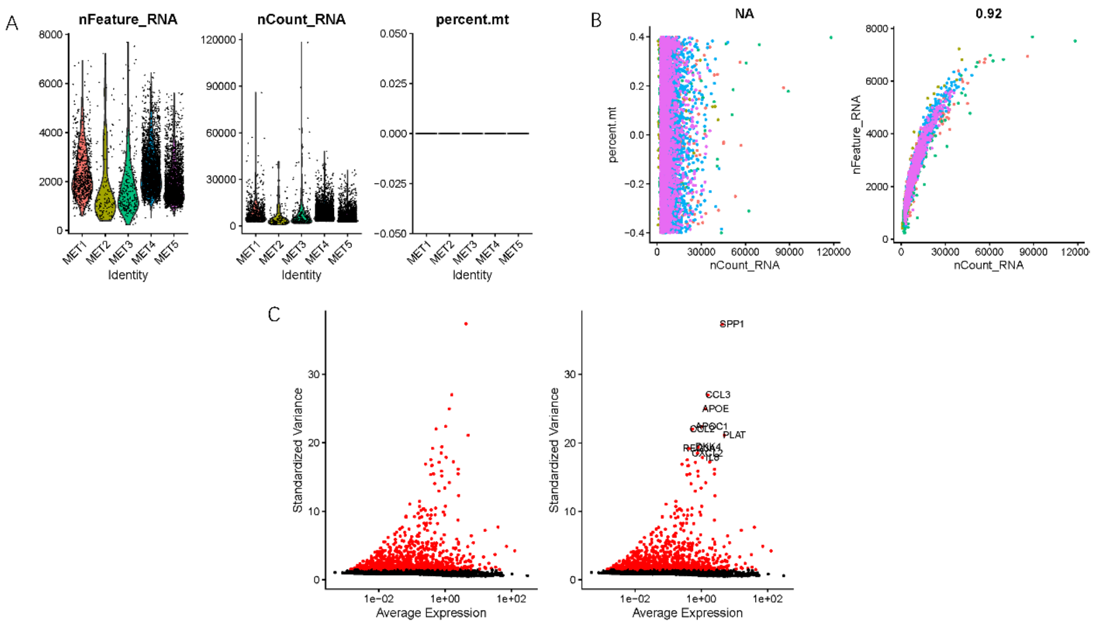

Supplementary Figure 1: Characterization of single-cell RNA sequencing from 121 cells and screening of marker genes. (A-B) Quality control of scRNA-seq for three cell sub-populations. We filtered out the cells with poor quality and analyzed the positive associations between detected gene counts and sequencing depth. (C) we identified the gene symbols with significant difference across cells and drawn the characteristic variance diagram.

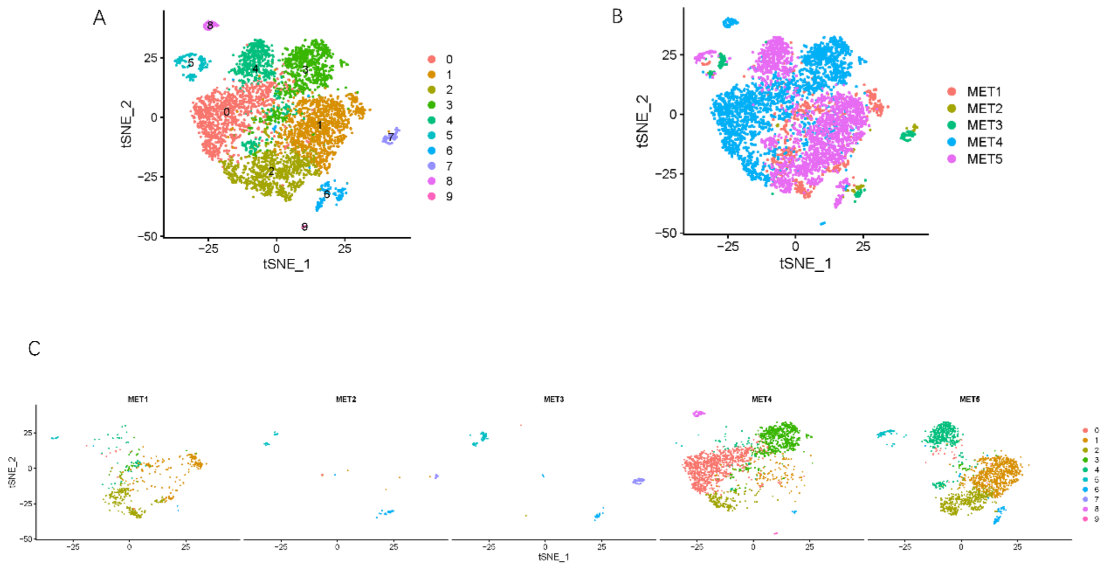

Supplementary Figure 2: Intratumoral heterogeneity of PDCA cells in 5 different metastatic lesions. (A) Ten main cellular subclusters were identified by t-SNE analysis. (B) t-SNE plot showing the 10 cell subclusters from 5 PDAC samples. (C) t-SNE plot showing the 10 cell subclusters under different samples.

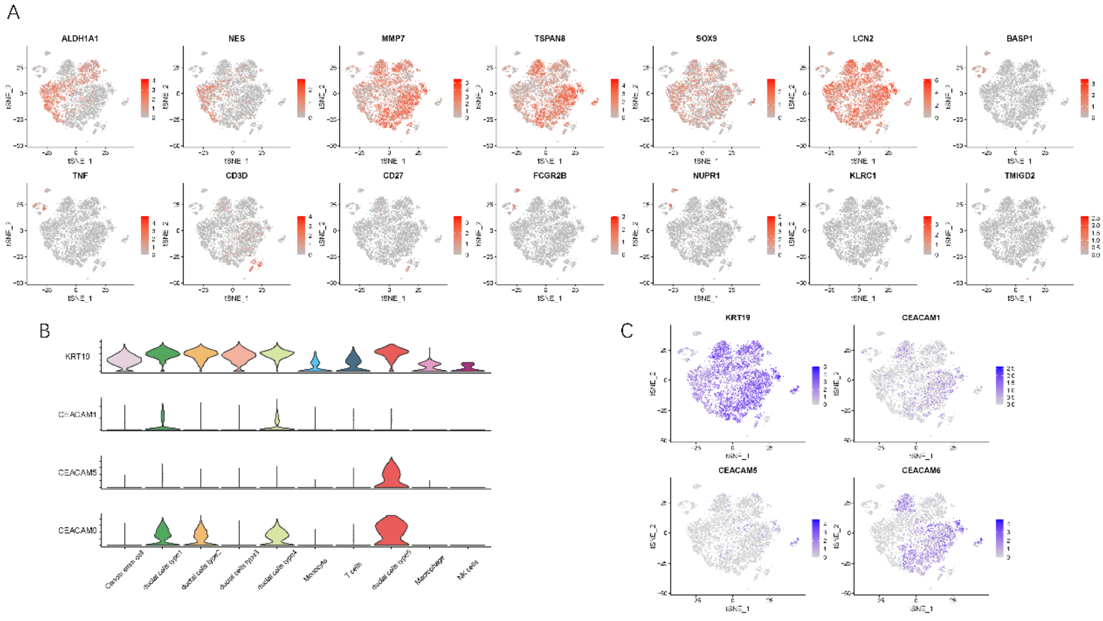

Supplementary Figure 3: (A) Expression levels of representative markers for cell subclusters are plotted onto the t-SNE map. Color key from gray to red indicates relative expression levels from low to high. The “expression level” was normalized by logNormalize method in Seurat. (B) Violin plots showing representative ductal markers are plotted onto the t-SNE map. (C) Feature plots showing representative ductal markers are plotted onto the t-SNE map.

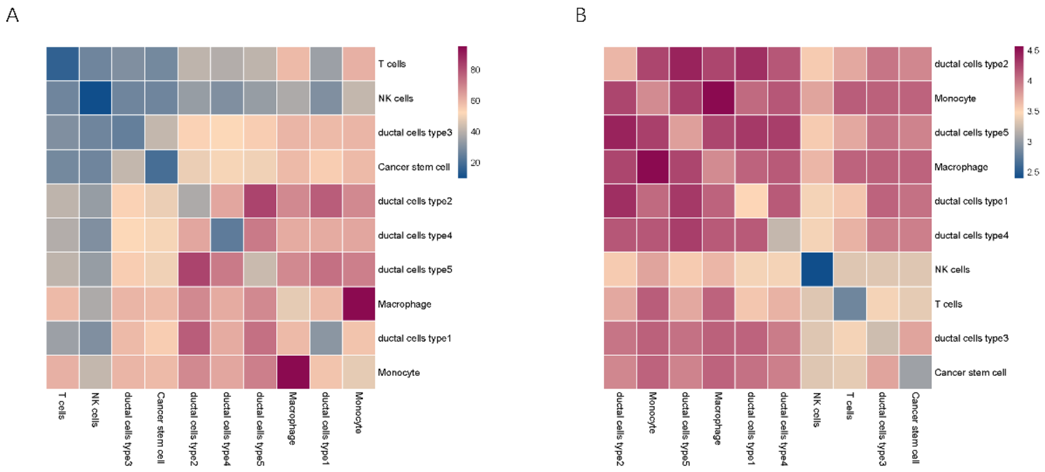

Supplementary Figure 4: The heatmap plot function of cell-cell interaction between tumor cells and immune cells. (A) under count value. (B) under log-count value. Color key from blue to red indicates relative expression levels from low to high.

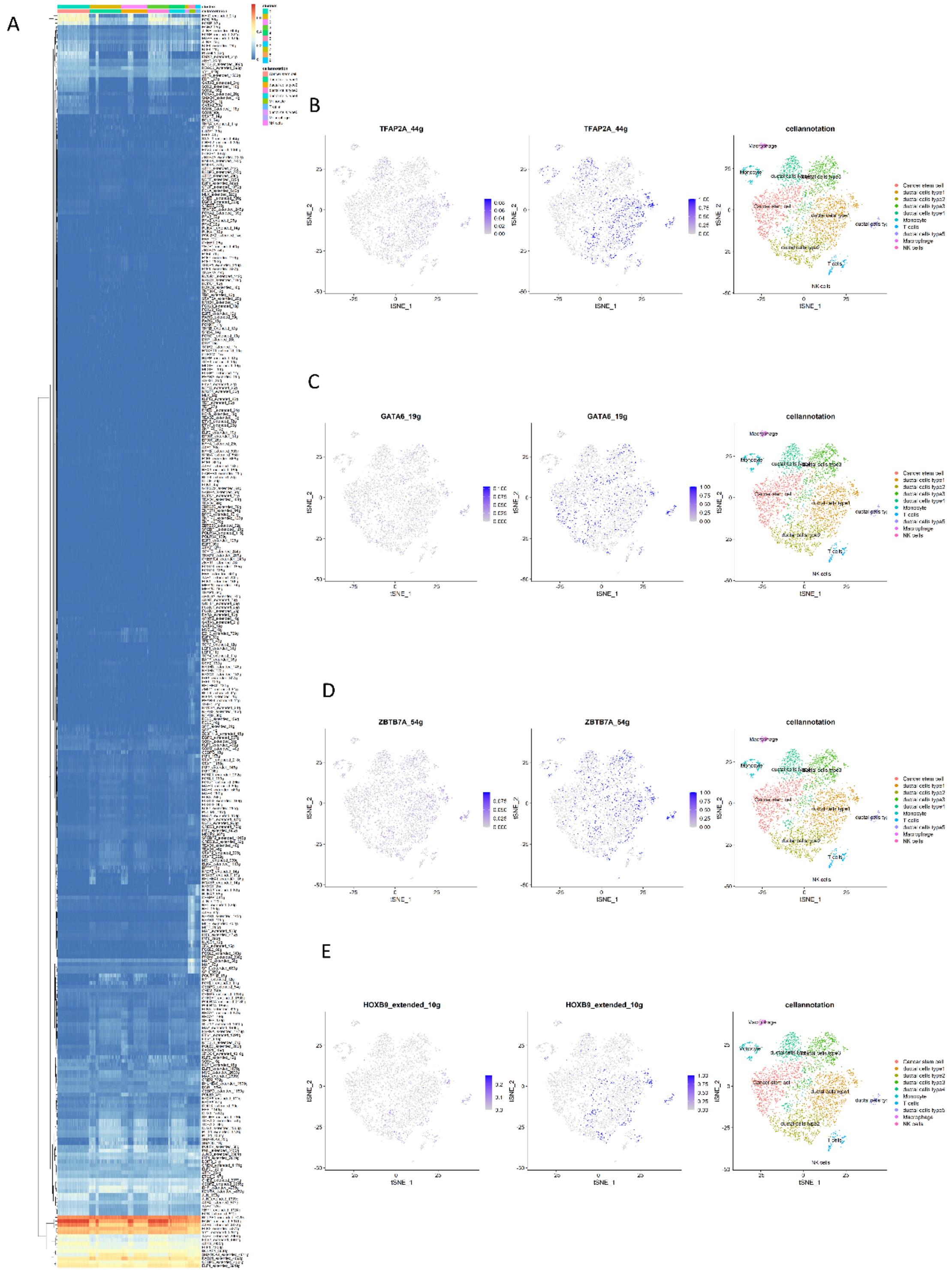

Supplementary Figure 5: Potential transcription factors involved in tumor progression (A) Heatmap of regulon activity analyzed by SCENIC. The t-SNE plot shows the top four highly expressed transcription factors in type 5 ductal cells and the corresponding downstream target genes. (B) TFAP2A. (C) GATA6. (D) HOXB9. (E) ZBTB7A.
